# Penetrating chest trauma secondary to falling on metallic (iron) bar

**DOI:** 10.4103/1817-1737.56005

**Published:** 2009

**Authors:** Hamid Al-Sayed, Hasan Sandogji, Abdullah Allam

**Affiliations:** *Department of Thoracic Surgery, King Fahad Hospital, Almadinah Almunawarah, Medina, Saudi Arabia*

**Keywords:** Haemothorax, chest injury, metalic bar

## Abstract

Case of a 27-year-old man who sustained penetrating chest injury caused by a metallic (iron) bar projecting from a pillar of a construction after he fell down from a height.

Thoracic and thoracoabdominal penetrating wounds present a frequent and challenging problem. The majority of these injuries can be managed nonoperatively. The selection of the patients for operation or observation can be made by clinical examination and appropriate investigations.[1] Penetrating chest trauma is frequently caused by gunshot and non—gunshot-related incidents such as stabs, traffic accidents and impalements.[2]

Rare penetrating chest injury by kebab's skewer,[3] hockey stick,[4] or self-inflicted with 5 knives[5] has been reported. We present here a very rare case of a 27-year-old man who sustained penetrating chest injury caused by a metallic (iron) bar projecting from a pillar of a construction after he fell down from a height.

## Case Report

A 27-year-old male construction worker presented to the emergency room of King Fahad Hospital after falling down, during working hours, from a height on a metallic (iron) bar projecting from a pillar of a construction.

In the emergency room, the patient was conscious, oriented, and stable hemodynamically. Clinical examination showed metallic (iron) rod with entry site at the right lumbar region and exit site at the left supraclavicular region, with no other external injury, fairly equal bilateral air entry, audible heart sound and no added sound, no raised JVP, flat and soft abdomen with minimal tender right hypochondrium, palpable peripheral pulses, and with all limbs moving.

**Figure 1:**
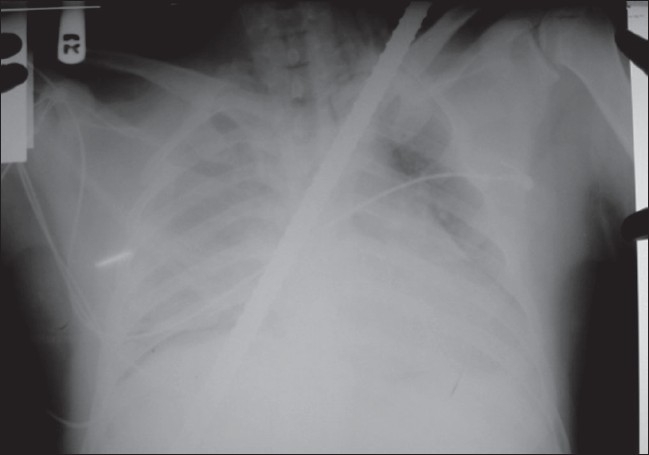
Chest X-ray showing metallic bar

The patient's oxygen saturation was maintained by a 5-liter oxygen face mask, and he was resuscitated with intravenous fluid through two large bore cannulas. Chest x-ray [Figure 1] was done, which showed a metallic rod traversing the chest cavity from right basal to left apical region through the mediastinum, with no obvious fracture of bone, pneumothorax or hemothorax, no widening of mediastinum and no cardiomegaly. Blood work-up showed no significant changes.

CT scan of the chest, abdomen and pelvis showed rounded metallic bar penetrating the right lumbar region, the posterior segment of the right hepatic lobe, right diaphragm, lower lobe of the right lung, posterior mediastinum, apex of left lung, and left supraclavicular fossa; also there was bilateral hemothorax, more on the right, and surgical emphysema on the left side.

**Figure 2:**
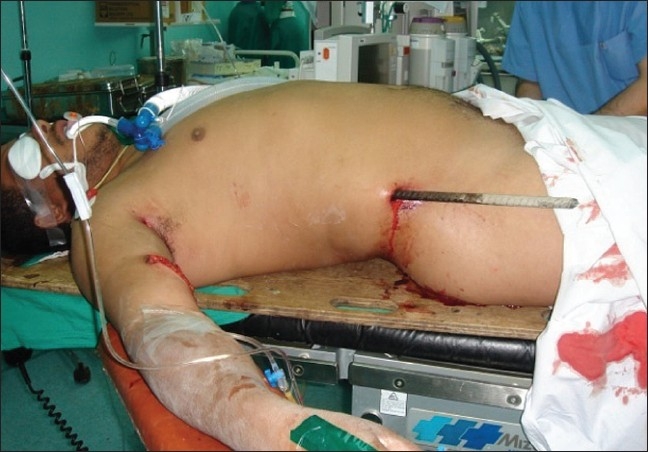
Entry point of the penetrating metallic bar

**Figure 3:**
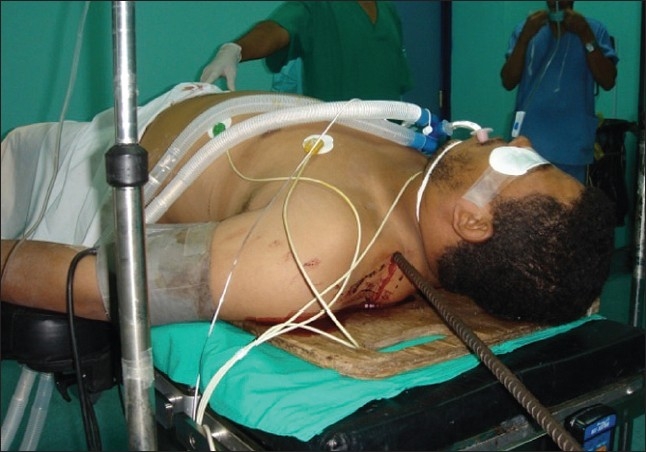
Exit point of the penetrating metallic bar

**Figure 4:**
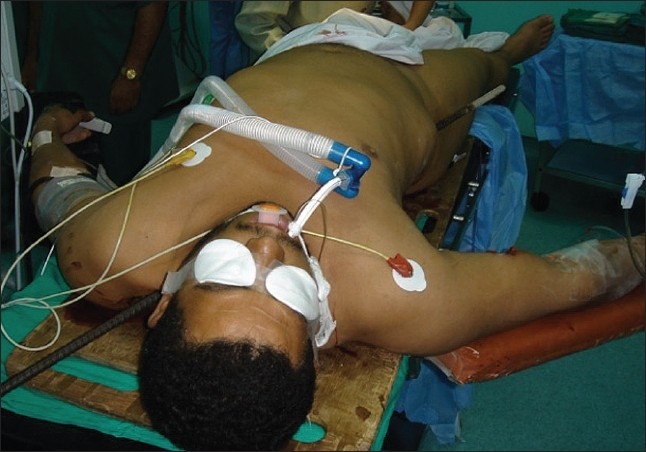
Entry point and exit point of the penetrating metallic bar

The patient was transferred to the operating room, where exploration laparotomy and right posterolateral thoracotomy was performed [Figures 2–4]. The metallic bar was pulled out ‘helically.’ The injury of the lower lobe of the right lung was repaired using 3-0 polypropylene to control the air leak. The right diaphragmatic injury was repaired using 1 polypropylene. The right hepatic lobe injury was repaired and hemostasis was controlled. Intraoperative esophagoscopy showed no esophageal injury. Bilateral chest tubes were inserted and the surgical wounds were closed into layers; and the patient was shifted to intensive care unit (ICU), where the course went smoothly for 2 days, apart from right surgical emphysema, which was controlled by suction applied to the right chest tube.

The patient was treated by broad-spectrum antibiotics and weaned from mechanical ventilation. He was then discharged from ICU and admitted to the ordinary ward, after which he had uneventful recovery and was discharged from the hospital in good general condition.
